# Iatrogenic botulism: a risk for botulinum toxin's medical use?

**DOI:** 10.1007/s00702-025-03083-y

**Published:** 2026-01-31

**Authors:** Dirk Dressler, Jürgen Frevert, Eric A. Johnson, Klaus Fink, Sabine Pellett, Sanjay Pandey, Uwe Walter, Pawel Tacik, Petr Kanovsky, Gholam Ali Shahidi, Norbert Brüggemann, Raymond L. Rosales, Maja Relja, Lingjing Jin, Jose Alberto Sagastequi Rodriguez, Lizhen Pan, Gerard E. Francisco, Huifang Shang, Xue Bai, Fereshte Adib Saberi

**Affiliations:** 1 Movement Disorders Section, Department of Neurology, Hannover Medical School, Carl-Neuberg-Str. 1, 30625 Hannover, Germany; 2 Botulinum Toxin Research Center, Tongji University School of Medicine, Shanghai, China; 3 Merz Pharmaceuticals, Frankfurt/M, Germany; 4 Department of Bacteriology, University of Wisconsin-Madison, Madison, WI USA; 5 Department of Neurology and Stroke Medicine, Amrita Institute of Medical Sciences, Amrita Vishwa Vidyapeetham, Faridabad, India; 6 Department of Neurology, German Center for Neurodegenerative Diseases (DZNE) Rostock/Greifswald, Rostock University Medical Center, Rostock, Germany; 7 Department of Parkinson’s Disease, Sleep and Movement Disorders, University Hospital Bonn, Bonn, Germany; 8 Department of Neurology and Clinical Neuroscience Center, Faculty of Medicine and Dentistry, University Hospital Olomouc, Palacky University, Olomouc, Czechia; 9 Department of Neurology, Firoozgar Hospital, Iran University of Medical Sciences, Tehran, Iran; 10 Department of Neurology, Lübeck University, Lübeck, Germany; 11 Research Center for Health Sciences, University of Santo Tomas, Manila, Philippines; 12 Medical School, University of Zagreb and Croatian Academy of Medical Sciences, Zagreb, Croatia; 13 Department of Neurology and Neurological Rehabilitation, Shanghai Yangzhi Rehabilitation Hospital, Tongji University School of Medicine, Shanghai, China; 14 Department of Neurology, University of Monterrey, Monterrey, Nueva Leon Mexico; 15 Department of Neurology, Tongji Hospital, Tongji University School of Medicine, Botulinum Toxin Research Center, Tongji University School of Medicine, Shanghai, China; 16 Physical Medicine and Rehabilitation, UTHealth McGovern Medical School, TIRR Memorial Hermann Hospital, Houston, TX USA; 17 Laboratory of Neurodegenerative Disorders, Department of Neurology, National Clinical Research Center for Geriatrics, West China Hospital, Sichuan University, Chengdu, China; 18 Department of Neurology, Shanxi Provincial People’s Hospital, Taiyuan, China; 19 IAB - Interdisciplinary Working Group for Movement Disorders, Hamburg, Germany

**Keywords:** Botulinum toxin, Therapeutic use, Iatrogenic botulism, Systemic spread, Adverse effects

## Abstract

Botulinum toxin (BT) is infamous for its extreme toxicity. If it enters the bloodstream, it can cause botulism presenting with a typical pattern of motor and autonomic dysfunction. An international expert panel organised by IAB—Interdisciplinary Working Group for Movement Disorders explored iatrogenic botulism after BT's medical use (IB), reached conclusions and formulated recommendations. When injected into its target tissue, BT binds to gangliosides on cholinergic nerve terminals before it is internalised permanently. Small amounts of BT, however, are circulating within the bloodstream. When BT type B is applied, IB-B occurs frequently, typically affecting the autonomic nervous system. When BT type A is applied, IB-A only occurs in special circumstances, even when high doses are used. We identified 236 patients with IB-A in the literature. All IB-A was mild or moderate and fully reversible. In 212 patients, it occurred with unapproved BT use. In 116 of them, unapproved BT preparations were used, in 81, unapproved indications were treated and in 15, underlying neuromuscular impairment including myasthenia gravis, Lambert-Eaton myasthenic syndrome, amyotrophic lateral sclerosis and spinal muscle atrophy were contraindications for BT use. In 24 patients, IB-A occurred in approved BT use. Their evaluation was frequently incomplete, so that causes for IB-A often remain unclear. They may include presence of differential diagnosis, subclinical neuromuscular impairment and interference with additional diseases. When IB is suspected, proper evaluation is necessary to verify it and to identify its causes. Off-label use is common in BT therapy. However, it should be performed with caution, especially in children and when high doses are applied. High BT doses should not be applied to low volumes of target tissues, in order not to exceed the BT binding capacity.

## Introduction

Botulinum toxin (BT) is infamous for its extreme toxicity. If it enters the bloodstream, it can cause botulism, presenting with a typical pattern of motor and autonomic impairment. In severe cases, critical illness with respiratory arrest and autonomic collapse may occur. In mild cases, however, it may remain undiagnosed. When used medically, either for therapeutic or for aesthetic purposes, the risk of iatrogenic botulism (IB) has to be addressed. Under normal circumstances, IB is avoided by BT's binding to its target tissues and by application of minute BT doses. However, IB has been reported in BT's medical use. We wanted to explore, how and when this may occur and what conclusions and recommendations may be drawn.

## Methods

This paper is based on a consensus initiative organised by IAB—Interdisciplinary Working Group for Movement Disorders, a multidisciplinary special interest group promoting multimodal and interdisciplinary therapies in movement disorders including BT therapy. IAB is a charitable organisation based in Hamburg, Germany. It has a world-wide reach and is independent from the pharmaceutical industry.

Publications on IB after BT therapy were identified by a literature search using PubMed (National Center for Biotechnology Information, National Library of Medicine, National Institutes of Health) with a wide scope of search words including 'botulinum toxin therapy', 'botulism', 'iatrogenic', 'systemic spread', 'systemic adverse effects', 'myasthenia gravis', 'Lambert Eaton myasthenic syndrome', 'amyotrophic lateral sclerosis', 'antitoxin', 'electrophysiological abnormalities', 'spinal neuromuscular atrophy', 'general weakness', 'electrodiagnostic evaluation', 'Black Box Warning', 'antitoxin'. Given the rarity of IB, the retrieval process was optimised to retrieve as many publications of interest as possible. All publications retrieved were screened for relevance. Cross-referencing further consolidated the publication data base, which was used by a publication committee (DD, FAS, JF, EAJ) to provide a manuscript draft which was circulated to an international panel of BT experts, modified according to the experts' comments and then approved by each expert.

Publications on IB after use of BT type A (IB-A) were classified as follows: 1) IB-A with use of unapproved BT drugs, 2) IB-A with use of BT in unapproved indications: 3) IB-A in the presence of contraindications 4) IB-A with approved BT therapy. In some cases, BT therapy was unapproved in more than one aspect. If this occurred, use of unapproved BT drugs was used for classification. In some cases, information on BT therapy was missing or ambiguous. If this occurred, classification was based on likelihoods at the discretion of the publication committee.

### General safety profile of botulinum toxin

Despite its extreme toxicity, BT has an extremely benign safety profile when used in medicine properly. After injection into the target tissue, BT binds to gangliosides on cholinergic nerve endings, before its light chain is rapidly transported intracellularly by a specific transport process using the acetylcholine vesicle recycling mechanism. Here, it stays until it is metabolised. With its local application and remain, BT avoids typical absorption, excretion and metabolic problems. Its effects are fully reversible. Cumulative effects have not been observed, although BT is typically applied for prolonged periods of time when used medically. Direct central nervous system effects above the spinal level have not been detected in patients so far.

BT's medical use may produce adverse effects. Inherent adverse effects are unavoidable. They are directly related to BT's mode of action. When a target muscle is injected to reduce unwanted muscle activity as in dystonia, spasticity or tremor, the target muscle's voluntary muscle activity is reduced by the same amount. Local and regional adverse effects are caused by BT spread into tissues adjacent to the primary target tissue. BT spread can be detected clinically, but also in animals by using histological techniques detecting glycogen-depletion (Shaari et al. 1991), acetylcholine esterase (Borodic et al. 1990) and muscle fibre atrophy (Borodic et al. 1994). Local and regional adverse effects are mainly determined by the indication BT is used for. The only cases, when BT's medical use had lethal effects, were cases of local and regional spread, initiating a chain of events including medical malpractice that indirectly produced secondary complications. Systemic adverse effects, or IB, are localised so far away from the injection site, that BT transport within the bloodstream is required.

### Definitions

Systemic spread describes the wide-spread distribution of BT within an organism. It is caused by BT entering the bloodstream. Systemic spread is assumed, when BT effects can not be explained by local spread around the BT injection site. When systemic spread produces systemic adverse effects, the term botulism is used. In IB, the medical use of BT, either for therapeutic or for aesthetic purposes, is the cause of botulism. IB-A is used, when BT-A was applied, IB-B, when BT-B was applied.

### Systemic spread of botulinum toxin (BT)

Systemic spread of BT in humans can be detected by clinical examinations and by single fibre electromyography (SF-EMG), a highly sensitive neurophysiological method to detect jitter, ie the occurrence of irregular neuromuscular transmission as a typical sign of botulism. Additionally, autonomic testing, particularly heart rate variability measurements, can detect systemic spread of BT. In animals, systemic spread can also be explored by radioactive-labelling of BT (Tang-Liu et al. 2003).

When BT-A is applied medically, systemic spread occurs regularly and can be detected by SF-EMG, even when low or intermediate BT doses are used (Garner et al. 1993; Girlanda et al. 1992; Lange et al. 1991, Olney 1988). Autonomic testing also may reveal systemic BT effects, even when low BT doses are applied (Girlanda et al. 1992). When BT-B is applied medically, clinically relevant systemic spread was suspected soon after its introduction as NeuroBloc^®^ Myobloc^®^ (Racette et al. 2002). In 2003, IB-B was first described, that BT-B frequently produces IB-B, even when low to moderate BT-B doses are applied (Dressler & Benecke 2003). IB-B is typically associated not with motor impairment, but with autonomic impairment (Dressler and Benecke 2003). Subsequent studies confirmed these findings (Rossi et al. 2006, Partikan et al. 2007, Racette et al. 2002).

### Iatrogenic botulism after application of botulinum toxin type A

When large patient cohorts are treated with BT-A, no motor or autonomic IB-A is detected, even when high and very high doses of up to Xeomin^®^ 1250MU are applied in spasticity and dystonia (Dressler et al. 2015; Wissel et al. 2017).

Under special circumstances, however, IB-A may occur. Until now, 236 cases of IB-A have been reported in the literature. In 212 cases, BT use was unapproved, because unapproved drugs were used or because unapproved indications were treated or because contraindications for BT use were present. In some cases, several violations of approved use occurred.

*IB-A with use of unapproved BT drugs:* As shown in Table 1, a total of 116 cases of IB-A occurred with the use of unapproved BT preparations. With two exceptions, IB-A was mild to moderate and transient. Unapproved BT preparations included BT drugs without approval in major Western countries, counterfeit BT drugs and even BT not intended for human use. In the infamous Florida cases of 2004, research-grade BT was applied for aesthetic use in massive overdoses in 4 patients (Chertow et al. 2006). IB-A provoked was dramatic and life-threatening and patients had to be treated under intensive care conditions for months. In another case from New Jersey, research-grade BT had been applied for aesthetic use also in massive doses producing severe and prolonged IB-A (Souayah et al. 2006). In China, 95 cases of IB-A were reported, where counterfeit BT drugs have been applied for various aesthetic indications (Bai et al. 2018; Li et al. 2018). In Egypt, 9 patients including children developed IB-A after receiving an unapproved BT drug called Neuroxin^®^ for infantile cerebral palsy, spasticity and dystonia (Monaem et al. 2018). In Turkey, 5 patients developed IB-A after receiving unapproved or unknown BT drugs to induce gastroparesis for weight loss as an unapproved indication and for axillary hyperhidrosis (Eser et al. 2024). In Switzerland, 2 patients developed IB-A after receiving unknown BT drugs for gastric paresis (Juillerat et al. 2024). When unapproved BT preparations were used, overdosing due to a lack of proper potency labelling may have occurred. When approved BT drugs were used, BT doses applied were, per se, not massive, but may have exceeded the stomach's binding capacity for BT, a concept originally introduced by Dressler (Dressler et al. 2015).

**Table 1:** 116 cases of iatrogenic botulism with use of unapproved botulinum toxin preparations

Authors Year	Number of patients	Indication	Target muscles	Total dose	Remarks
Chertow et al. 2006 Florida cases of 2004	4	Aesthetic	Facial muscles	> 8Mio MU	Laboratory-grade BT
Souayah et la. 2006 New Jersey case of 2006	1	Aesthetic	Facial muscles	?	Laboratory-grade BT
Bai et al. 2018 China cases-1 of 2008	86	Aesthetic	Facial muscles Calf muscles Masseter muscles Others	?	Counterfeit BT
Li et al. 2018 China cases-2 of 2008	9	Aesthetic	Facial muscles	?	Counterfeit BT
Rashid et al. 2018 Egypt cases of 2018	9	ICP (7) Spasticity (1) Hyperhidrosis (1)	Leg muscles Arm muscles Axilla	N200-300 (5) N? (3) unknown (1)	Neuroxin® (8) Unknown (1) Mostly children
Eser et al. 2024 Turkey cases of 2024	2 3	Hyperhidrosis Gastric paresis	Axilla-BL Stomach	? ?	Unapproved BT (2) Unknown BT (3) Additional case reported in Table 2
Juillerat et al. 2024	2	Gastric paresis	Stomach	?	

*BT* unspecified botulinum toxin, *D[X]* Dysport® [dose in mouse units], *N[X]* Neuroxin® [dose in mouse units],* ICP* infantile cerebral palsy

? values not reported

*IB-A with use of BT in unapproved indications:* As shown in Table 2, a total of 81 patients with IB-A with use of BT in unapproved indications have been reported so far. Usually, IB-A was mild to moderate and transient. In Turkey, in 2023 a large number of patients developed IB-A after receiving BT-A to induce gastric paresis for weight loss (Islam et al. 2023; Dorner et al. 2023; Eser et al. 2024; Juillerat et al. 2024). Some patients received approved BT drugs, others received unapproved BT drugs. As most of these patients were visiting Turkey for this procedure, reports appeared from throughout Europe (Dorner et al. 2023; Juillerat et al. 2024). In Korea, IB-A occurred after unapproved aesthetic calf injections with an unspecified BT drug (Fan et al. 2016).

**Table 2:** 81 cases of iatrogenic botulism after botulinum toxin use in unapproved indications

Authors Year	Number of patients	Indication	Target muscles	Total dose	Remarks
Fan et al. 2016	1 1	Calf contouring Calf contouring Facial aesthetics	Calf muscles-BL Calf muscles-BL Facial muscles-BL	B500? B400?	antitoxin with questionable effect
Dorner et al. 2023	78	Gastric paresis	Stomach	B?/D?	Turkey (53) Germany (21) Switzerland (2) Austria (1) France (1)
Eser et al. 2024 Turkey cases of 2024	1	Gastric paresis	Stomach	D1000	Additional cases reported in Table 1

*BT* unspecified botulinum toxin, *BL* bilateral, *B[X]* Botox® [dose in mouse units], *N[X]* Neuroxin® [dose in mouse units]

? probably/not known

*IB-A in the presence of contraindications:* The use of BT therapy is contraindicated in the presence of neuromuscular disorders increasing the patient's sensitivity against BT and, thus, generating IB-A. As shown in Table 3, a total of 15 cases of underlying neuromuscular disorders have been described so far including myasthenia gravis (Emmerson et al. 1994, Tarsy et al. 2000; Fasano et al. 2005; Iwase & Iwase 2006; Dressler 2010; Watts et al. 2015; Hacer et al. 2016, El-Heiss et al. 2017, Timmermann et al. 2019), Lambert-Eaton myasthenic syndrome (Erbguth et al. 1993), amyotrophic lateral sclerosis (Kent et al. 2013, Mezaki et al. 2016) and spinal muscular atrophy type 1 (Kim et al. 2022). All cases have been mild to moderate. IB-A in muscular diseases has not been reported so far. BT therapy in myotonia congenita did not produce IB-A (Dressler and Adib Saberi 2014). Appropriate BT dose reductions allow to apply BT therapy in the presence of contraindications (Goncalves et al. 1999, Dressler 2010, Elvarasi and Goyal 2021).

**Table 3:** 15 cases of iatrogenic botulism after botulinum toxin type A use in contraindicated conditions

Authors Year	Number of patients	Indication	Target muscles	Total dose	Remarks
*Myasthenia gravis*					
Emmerson 1994	1	Cervical dystonia	Neck muscles-BL	D300	
Brunnschweiler 1997	1	Cervical dystonia	Neck muscles-BL	D?/B?	
Tarsy et al. 2000	1	Meige syndrome	Facial muscles-BL	B120	
Fasano et al. 2005	1	Cervical dystonia	Neck muscles-BL	B120	
Iwase and Iwase 2006	1	Cervical dystonia blepharospasm	Neck muscles-BL Periocular muscles-BL	B180	
Dressler 2010	1	Cervical dystonia	Neck muscles-BL	B110-180	B72: no AE
Watts et al. 2015	1	Epiphora	Lacrimal gland	D6	
Hacer et al. 2016	1	Oromandibular dystonia	Hypoglossal muscle-BL	B20	
El-Heiss et al. 2017	1	Hyperhidrosis	Axilla-BL	D100	
Timmermann et al. 2019	1	Cosmetic	Facial muscles-BL	D84	
Elavarasi and Goyal 2021	1	Hemifacial spasm	Facial muscles-UL	B24?	Diplopia only
*Lambert-Eaton myasthenic syndrome*					
Erbguth et al. 1993	1	Blepharospasm	Periocular muscles-BL	PP12	
*Amyotrophic lateral sclerosis*					
Kent et al. 2013	1	Spasticity	Calf muscles-UL	D1000	Cerebellar haemorrhage
Mezaki et al. 2016	1	?	?	D?/B?	
*Spinal muscular atrophy*					
Kim et al. 2022	1	Hypersalivation	Parotid gland-BL Submandibular gland-BL	B7	Age 17 months

*UL* unilateral, *BL* bilateral, *B[X]* Botox® [dose in mouse units], *D[X]* Dysport® [dose in mouse units], *PP[X]* Porton Products® [dose in ng]

? value not reported, probable value

*IB-A with approved BT therapy:* As shown in Table 4, 24 cases of IB-A with approved use (approved BT drugs, approved indications) have been reported so far. All cases have been mild to moderate and transient. 14 Patients were treated for spasticity, 5 for dystonia and 5 patients for autonomic indications. Dysport® and Botox® were equally often used. BT doses were robust, but within recommendations. In one case report (Ghasemi et al. 2012), low dosed Botox® was used for axillary hyperhidrosis (30MU total dose) and palmar hyperhidrosis (30MU total dose). All other treatment parameters were unremarkable. Evaluation of IB-A was often incomplete: Without SF-EMG, the assumed presence of BT was not proven and without neurophysiological testing, subclinical neuromuscular impairment may be possible. Cross-reactions with coexisting disorders and atypical allergic reactions, like flu-like symptoms, may have mimicked IB-A. Psychological reactions may be IB-A lookalikes, especially when the clinical symptomatology is mild.

**Table 4:** 24 cases of iatrogenic botulism with approved use of botulinum toxin

Authors Year	Number of patients	Indication	Target muscles	Total dose	Remarks
*Spasticity*					
Bakheit et al. 1997	2	Spasticity Spasticity	Thigh muscles-UL Neck muscles	D250 D250	Multiple sclerosis Multi-system atrophy
McMahon 2002	1	Spasticity	Calf muscles-BL	B?/D?	Infantile cerebral palsy
Goldstein 2006	1	Spasticity	Leg muscles-BL	B1000	Infantile cerebral palsy Age 13 years
Duffey and Brown 2006	1	Spasticity	Calf muscles-BL	D1000	
Howell et al. 2007	1	Spasticity	Leg muscles-BL	B400	Infantile cerebral palsy Age 9 years
Varghese-Kroll and Elovic 2009	1	Spasticity	Arm muscles-UL Leg muscles-UL	B800	Stroke
Coban et al. 2010	3	Spasticity Spasticity Spasticity	Calf muscles-BL Leg muscles-BL Calfmuscles-BL	D1500 D1500 D1500	Hereditary spastic paraparesis Infantile cerebral palsy Herditary spastic paraparesis
Crowner et al. 2010	3	Spasticity Dystonia spasticity	Arm muscles-UL Leg muscles-UL Axial muscles Arm muscles-UL Leg muscles-UL	B640 B760 B700	Stroke Tumor
Thomas et al. 2012	2	Spasticity Spasticity	Arm muscles-UL Leg muscles-UL Arm muscles-UL Leg muscles-UL	B700 B700	Stroke Stroke
*Dystonia*					
Bhatia et al. 1999	3	Cervical dystonia Hemidystonia Hemidystonia	Neck muscles Arm muscles-UL Leg muscles-UL	D650 D900 D600	
Szuch et al. 2017	1	Foot dystonia	Calf muscles-UL	B600	Parkinson's disease polyneuropathy
*Autonomic indications*					
Tugnoli et al. 2002	1	Hyperhidrosis	Axilla-BL Palm-BL	D1400	
Ghasemi et al. 2012	2	Hyperhidrosis Hyperhidrosis	Axilla-BL Palm-BL	B30 B30	
Wyndaele and VanDromme 2002	2	Neurogenic bladder Neurogenic bladder	Detrusor vesicae Detrusor vesicae	D1000 B300	

*UL* unilateral, *BL* bilateral, *B[X]* Botox® [dose in mouse units], *D[X]* Dysport® [dose in mouse units], ? value not reported, probable value

### Treatment

BT antitoxin may be given in botulism, including IB. With BT's rapid intracellular internalisation, it is unlikely, that BT antitoxin can neutralise significant amounts of BT. Reports did not demonstrate robust effects (Fan et al. 2016). Treatment should focus on stabilisation of impaired vital systems, including ventilation and autonomic functions. If dysphagia occurs, aspiration pneumonia should be prevented by application of a gastric tube and early application of adequate antibiosis. Food and fluid intake should be balanced.

### Evaluation

Table 5 shows an overview of the recommended evaluation of suspected iatrogenic botulism. The patient's history should confirm an adequate temporal relationship between the BT application and the onset of the clinical symptomatology. The temporal profile of the further course of the symptomatology should be compatible with the time course of the BT effect. The clinical examination should reveal the typical clinical symptomatology consisting of motor and autonomic impairment. SF-EMG demonstrates the typical neuromuscular jitter caused by BT. Autonomic testing may confirm autonomic impairment, even when the clinical symptomatology is mild. Serial muscle stimulation may detect underlying neuromuscular impairment and the presence of specific antibodies may demonstrate myasthenia gravis, even when it is subclinical.

**Table 5:** Recommended workup for suspected iatrogenic botulism

*History*
Onset in relationship to BT application
Temporal profile of symptomatology
Underlying and co-existing diseases?
*Clinical examination*
Motor symptoms
Autonomic symptoms
*Investigations*
Single fibre electromyography (SF-EMG)
Autonomic testing
Serial muscle stimulation
Myasthenia gravis antibodies

## Conclusions and recommendations

IB has to be an obvious concern, when BT is applied for medical use. When BT-A is used medically, systemic spread frequently occurs, even when moderate BT doses are applied. However, this spread is usually not associated with IB-A, even, when high BT-A doses in off-label settings are applied.

IB-A may occur in special circumstances. It is usually mild and always fully reversible. Special circumstances include the unapproved BT use with use of unapproved BT preparations, use in unapproved indications and the presence of contraindications for BT therapy. IB-A may also occur rarely in approved BT use. Reasons for this remain unclear, as often reported cases are not fully evaluated, so that underlying neuromuscular disorders, cross reactions with coexisting disorders, atypical allergic reactions and psychological reactions may be causative. When IB-A is caused by an underlying neuromuscular disease, dose adaptation allows to continue BT therapy. Caution should be applied when BT therapy is used off-label, especially in children. Dosing should then be done carefully. When IB is suspected, appropriate evaluation should confirm it and identify possible causes. When IB-A is confirmed, medical attention should be sought and symptomatic treatment should be applied where necessary.

IB is a minor risk for the medical application of BT. Fatalities after medical use of BT were not caused by IB, but by local effects producing dysphagia with aspiration pneumonia.

## Data Availability

No datasets were generated or analysed during the current study.
